# Gendered Expectations: Examining How Peers Shape Female Students' Intent to Pursue STEM Fields

**DOI:** 10.3389/fpsyg.2017.00329

**Published:** 2017-03-15

**Authors:** Catherine Riegle-Crumb, Karisma Morton

**Affiliations:** Department of Curriculum and Instruction, STEM Education, and Department of Sociology, University of TexasAustin, USA

**Keywords:** gender, computer science, engineering, peers, stereotypes, college

## Abstract

Building on prior psychological and sociological research on the power of local environments to shape gendered outcomes in STEM fields, this study focuses on the critical stage of adolescence to explore the potential negative impact of exposure to exclusionary messages from peers within girls' science classrooms, as well as the positive potential impact of inclusionary messages. Specifically, utilizing longitudinal data from a diverse sample of adolescent youth, analyses examine how the presence of biased male peers, as well as confident female peers, shape girls' subsequent intentions to pursue different STEM fields, focusing specifically on intentions to pursue the male-dominated fields of computer science and engineering, as well as more gender equitable fields. Results reveal that exposure to a higher percentage of 8th grade male peers in the classroom who endorsed explicit gender/STEM stereotypes significantly and negatively predicted girls' later intentions to pursue a computer science/engineering (CS/E) major. Yet results also reveal that exposure to a higher percentage of confident female peers in the science classroom positively predicted such intentions. These results were specific to CS/E majors, suggesting that peers are an important source of messages regarding whether or not girls should pursue non-traditional STEM fields. This study calls attention to the importance of examining both positive and negative sources of influence within the local contexts where young people live and learn. Limitations and directions for future research are also discussed.

## Introduction

While women in the U.S have made substantial progress in recent decades in many high-status areas, including comprising more than half of recent entering classes in both medical school and law school, and clearly surpassing their male peers in rates of college matriculation and attainment, nevertheless stark instances of inequality remain (DiPrete and Buchmann, 2013). Specifically, women remain substantially under-represented in some STEM fields, most markedly in computer science and engineering, two of the most in-demand and high-paying domains (National Academy of Sciences, 2007; England, 2010). Yet conditional on entry to college majors in these and other STEM fields, women persist to attain a degree at comparable rates to men (Xie and Shauman, 2003; Barton et al., 2008; Ohland et al., 2008). Thus, a principle barrier to equitable female representation among STEM degree earners, and subsequently, among STEM professionals, is the fact that females are much less likely than males to pursue these fields in college in the first place.

Additionally, it is important to recognize that the roots of gender inequality in the choice of college major reach further back. Research reveals that educational and occupational aspirations began to crystallize around early adolescence, coinciding with the increasing saliency of gender identity and gender roles in young people's lives (Bandura et al., 2001; Eccles, 2007; Eccles and Roeser, 2011). As they actively contemplate their possible futures, young people are subject to a multitude of messages from those around them regarding what is appropriate and expected for their gender. And at a developmental point when adolescents begin to move away from the parental sphere of influence, relationships with peers become increasingly salient, and thus signals and approval from them take on a newly powerful role (Wentzel et al., 2012) this may be even more pronounced for adolescent girls than boys, as they are socialized to be both more aware of and sensitive to others' opinions (Gilligan, 1982; Beutel and Marini, 1995).

Stepping back, it is clear that efforts to understand why young women are under-represented in certain STEM fields in college and beyond should focus on the formation of the gender gap in future educational and career plans that emerges during the adolescent years, and the critical role that peers play in this process (Eccles and Wigfield, 2002; Eccles, 2009). Indeed, scholars examining gender inequality in STEM fields have long acknowledged that peers and the classrooms and schools they populate likely play an important role in shaping young people's decisions (e.g., Eccles, 1994); yet nevertheless the bulk of prior literature on this topic has focused primarily on the impact of students' own attitudes and beliefs, such as self-efficacy and affect, as predictors of their subsequent STEM-related decisions (Eccles, 1994; Correll, 2001; Eccles and Wigfield, 2002).

Consequently, in this study, we focus needed attention on the role of peers in shaping adolescents' future intentions to pursue STEM fields, with the goal of understanding both positive and negative sources of influence on girls' decisions. Building on prior research in psychology and sociology, we examine the potential negative impact of exposure to exclusionary messages from peers within the local contexts of girls' science classrooms, as well as the positive potential impact of inclusionary messages. Specifically, informed by research on stereotype threat (Spencer et al., 1999; Inzlicht and Ben-Zeev, 2000), we examine whether being in a classroom where male students explicitly endorse gender/STEM stereotypes works to deter girls' intentions of pursuing STEM fields in the future. Additionally, building on the work of gender scholars who point to the power of counter-stereotypical evidence to disrupt gender norms (Dasgupta, 2011; Stout et al., 2011), we also examine whether being surrounded by female peers who are very confident in their science ability provides girls with messages of belonging and inclusion that subsequently promote their likelihood of pursuing STEM fields.

To investigate these issues, we utilize longitudinal data from middle and high school students collected in a large, urban, predominantly low-income and Hispanic school district in the U.S, a location that demographically mirrors the districts attended by millions of young people. Thus our study marks a departure from the majority of extant research on gender inequality in STEM that continues to focus on predominantly white populations despite both the changing demographics of the U.S. and the fact that minority students have relatively high levels of STEM interest (Hurtado et al., 2010; Riegle-Crumb et al., 2011; Xie et al., 2015). Our dataset is also relatively unique as it includes detailed information about peer attitudes and beliefs, which is made possible by surveying entire classrooms within schools. Additionally, we distinguish between future intentions to major in computer science and engineering, fields that are heavily male-dominated, compared to intentions to major in the biological and physical sciences, fields where women and men have similar rates of representation (Cheryan et al., 2017)^1^. In doing so we are better able to understand whether the exclusionary and inclusionary messages to which girls may be exposed to via their peers is particularly powerful in shaping their intentions to pursue fields that are non-normative for their gender.

## Background

Gender theorists and scholars argue that gender is a social construction, one that is created and reinforced across various levels, including the macro-level of institutions as well as the micro-level of the local environments that individuals inhabit (Ridgeway and Correll, 2004; Risman, 2004). Some scholars have argued that local environments are perhaps the most powerful location of the construction of gender, as the everyday interpersonal interactions that occur in homes, schools, and workplaces are where individuals first learn and are subsequently continually reminded of the normative expectations of others (Risman, 2004). Put simply, in the locations where girls and young women conduct their daily lives, they encounter a host of experiences and interactions with others who expect them to think and behave in ways that are consistent with prevailing societal gender norms and stereotypes. Yet at the same time, local environments offer the potential for the disruption of inequality and the creation of alternative constructions of gender (Deutsch, 2007; Risman, 2009). For example, if they are populated by individuals who do not endorse traditional norms and beliefs, local contexts can create opportunities for interactions and experiences that push back against larger social norms and paradigms.

Building on this logic, the schools that students attend and the classrooms within them are critical locations to investigate regarding the social construction of gender, and more specifically, the shaping of gendered expectations about STEM fields. A growing body of research in psychology and sociology has recognized and empirically examined how factors within local environments can recreate and reinforce traditionally gendered beliefs and roles, and to a considerably lesser extent, also considered how characteristics of local contexts can instead disrupt gender norms and stereotypes. Thus, below we briefly describe extant research on the power of exclusionary messages in local contexts to deter females' interest and pursuit of STEM fields, as well as research on inclusionary messages that can offer support for non-normative choices. As we articulate in more detail below, while certainly informative, the current literature is nonetheless limited in its ability to shed light on our understanding of inequality due to both substantive and methodological issues.

### Messages of exclusion within STEM local environments

Recent research on the topic of stereotype threat has investigated how gender stereotypes and bias can function within local environments to deter the STEM interest and achievement of females. This literature is largely experimental, with cues manipulated by researchers to assess how exposure to statements about female inferiority in math and science subsequently impacts the behavior and attitudes of female study participants (Spencer et al., 1999; Shapiro and Williams, 2012). Within this literature, some studies call specific attention to the role of male peers in invoking stereotypes, suggesting that the gender composition of the environment can be sufficient to invoke threat. Specifically, in environments where STEM performance is being somehow evaluated, those that are very male-dominated can activate the notion that females do not belong or are out of place, thus impacting subsequent behavior or attitudes (Inzlicht and Ben-Zeev, 2000; Murphy et al., 2007; Dasgupta et al., 2015). While research in this area has called critical attention to how proximate exposure to bias and stereotypes can negatively impact females' STEM-related outcomes, nevertheless it is important to point out the results of such experiments may not necessarily translate outside these highly controlled settings.

Beyond the experimental literature on stereotype threat, some studies have attempted to measure the impact of bias and stereotypes on deterring females in STEM fields by asking young women to recount their exposure to such factors. For example, a recent study by Brown and Leaper (2010) found that female students' perceptions of academic sexism, measured as the frequency of overt comments they recall hearing others make regarding female inferiority in math, was negatively related to their interest in pursuing STEM fields. This study did not however, differentiate between comments made by peers, teachers, or family members, thus obscuring the source of negative messages. Other studies, mostly qualitative, ask female college students in STEM fields to report the extent to which they have experienced being a target of discriminatory acts in the classroom (Seymour and Hewitt, 1997; Ecklund et al., 2012). Importantly these studies highlight that exclusionary interactions typically occur with male individuals, which is consistent with other research that finds that males are much more likely to endorse gender/STEM stereotypes than females (Schmader et al., 2004). Thus, extant research generally falls short of directly assessing the stereotypic beliefs held by peers in real-world settings, instead relying on individuals' recall of particular discriminatory events. While certainly important, this focus likely underestimates the extent to which females interact daily with biased male peers in local STEM environments in ways that may work subtly but cumulatively to deter their STEM interest.

Stepping back, we suggest that prior research on the power of exclusionary messages within STEM environments is informative but nonetheless limited. In addition to the reasons outlined in the preceding paragraphs, we also note that research in this area typically focuses on college-age students, including those already enrolled in STEM fields, rather than examining the impact of exclusionary messages at the more formative stage of adolescence. Thus our longitudinal study will contribute new knowledge to the field by explicitly examining whether the actual presence of gender-biased male peers in science classrooms deters the future STEM intentions of adolescent females.

### Messages of inclusion within STEM local environments

Given the continued prevalence of stereotypes about females' presumed innate inferiority in math and science domains, it is logical to assume that most (if not all) females live and learn in local environments in which they interact with biased individuals. Yet at the same time, it is possible that some local contexts serve to disrupt these larger gender stereotypes. In this vein, some psychological research has recently moved to empirically examine the power of peers and role models to counter-act stereotypes and provide alternative depictions of females' strength and belonging in STEM fields (Dasgupta, 2011). Sometimes referred to as the stereotype-inoculation model, researchers have found that exposure to female peers and adult role models whose own behaviors and accomplishments in STEM fields contradicts larger stereotypes can therefore increase young women's own sense of identification with STEM fields, and in doing so promote their own subsequent choices (Dasgupta and Asgari, 2004; Lenton et al., 2009; Stout et al., 2011). This mostly experimental body of research thus provides empirical evidence suggesting that peers can be the source of messages of inclusion within STEM-focused local environments.

Consistent with this notion, other research in psychology and sociology has focused on the power of friends to act as a source of support and encouragement for girls' STEM-related decisions. Specifically, female friends who themselves work hard and succeed in STEM fields can help to create a local environment where girls feel included, increasing their desire to continue to pursue such fields. For example, Riegle-Crumb et al. (2006) found that girls' decisions to take advanced math classes in high school were influenced by the presence of such peers. Other studies provide similar evidence that having female peers who are STEM-focused provides legitimation to girls' own pursuits and promotes the sense of belonging to a community (Frank et al., 2008; Robnett and Leaper, 2013; Leaper, 2015).

While this relatively small body of research demonstrates the potential for peers to act as positive sources of influence in shaping girls' STEM expectations, we note that as with extant research on peers as a source of exclusionary messages, there are limitations. Few studies in this area utilize real-world settings, and those that do focus on peers' academic performance but not their actual STEM attitudes (Riegle-Crumb et al., 2006; Frank et al., 2008), or rely on individuals' perceptions of the attitudes of their peers rather than direct measurement (e.g., Robnett and Leaper, 2013). Further, while this research does sometimes focus on adolescents at formative stages in their STEM-related decision-making, it also typically focuses on close friends as a subset of peers. Yet foundational research on peer influence persuasively argues that the viewpoints and attitudes of friends may sometimes be less consequential than those of the larger group of peers in the local environment, as friends' support can often be taken for granted in a manner that the support of other peers cannot (Giordano, 2003). Therefore our study provides a new contribution to the literature by measuring how the science-related views of female peers in the classroom can potentially impact adolescent girls' subsequent STEM intentions.

## Current study

Our study builds on the insights of several different areas of prior research to investigate how factors within the local environment of students' science classrooms can work to either deter or promote girls' future STEM intentions. Specifically, we will examine both the potential for male peers' biased beliefs to create messages of exclusion, as well as the potential for female peers' science confidence to provide supportive messages of inclusion and belonging in STEM fields. In doing so, we build upon prior research on stereotype threat, as well as social psychological research on stereotype inoculation and sociological research on the influence of peers. Our study attempts to bridge these relatively distinct areas of research to create new knowledge about the simultaneous influence of exclusionary and inclusionary factors at a critically important point when adolescents' intentions to pursue (or not to pursue) STEM majors are crystallizing, as these early decisions are highly predictive of subsequent patterns of gender inequality in STEM degree attainment and labor force participation (Xie and Shauman, 2003; Morgan et al., 2013).

Additionally, it is important to point out that the majority of prior literature that examines the potential impact of local contexts on girls' STEM outcomes does so utilizing samples of predominantly white students. Such a focus is very limited and does not capture the changing demographics of the U.S., particularly for the student-age population (Ayscue and Orfield, 2016). Regarding gender patterns in STEM, national studies have documented relatively similar gaps in interest and attainment that favor men across different racial/ethnic groups (Hanson, 2006; Riegle-Crumb and King, 2010; Xie et al., 2015); yet of course these patterns do not mean that the obstacles and experiences that shape Hispanic females' STEM choices, for example, are necessarily similar to those that are most relevant for white females. Our study therefore aims to contribute to the small body of extant research that examines gender disparities in STEM among a diverse population, with an eye towards understanding how experiences of social inclusion and exclusion might matter for girls from non-dominant backgrounds.

Finally, we note that the prior research discussed above is limited in its attention to different STEM domains, typically focusing only on one field, or instead considering STEM in the aggregate. Yet at the baccalaureate level and beyond, some fields are severely male-dominated while others are not. Therefore, in an effort to better understand the power of peer beliefs and attitudes to shape girls' future intentions, in this study we consider their potential influence both on intentions to pursue strongly male-dominated STEM fields (e.g., computer science and engineering), as well as more gender equitable fields (e.g., biological and physical sciences). Thus, our study will address the following research questions:

1a. Does exposure to *biased male peers* in their science classrooms negatively impact the intentions of female students to pursue college degrees in *computer science and engineering*?
1b. Does such exposure similarly impact female students' intentions to pursue degrees in the *biological and physical sciences*?
2a. Does exposure to *confident female peers* in their science classrooms positively impact the intentions of female students to pursue college degrees in *computer science and engineering*?
2b. Does such exposure similarly impact female students' intentions to pursue degrees in the *biological and physical sciences*?

## Data and sample

For this study we utilize the Broadening Science in School Study (BSSS), a dataset collected from a very large urban school district (approximately 200,000 students) in the southwestern U.S. The district is predominantly Hispanic (more than 70%), and also serves a student population that is economically disadvantaged, with more than 75% of students in the district eligible for either free or reduced lunch. Additionally, 25% of students are classified by the district as Limited English Proficient (LEP).

The research team collected administrative data (including academic transcripts) as well as surveys from two cohorts of students in 18 middle schools in the district during the fall of their 8th grade year (2013 or 2014). Students were surveyed in their science classrooms with a response rate of almost 90% per school. Importantly for the purposes of our study, we were able to aggregate the responses of individuals in the same science classroom to create the measures of peer attitudes and beliefs (as well as other characteristics of the classroom) described below. Additionally, the team followed a sub-set of students and briefly surveyed them again after they transitioned into high school (*n* = 11 high schools; response rate of approximately 70% per school). This later survey is the source of our dependent variable (intentions to major in various STEM fields) described below.

Our final analytic sample is comprised of 1,273 high school students (647 females and 626 males) for whom 8th grade middle school administrative and survey data were available, who were not missing on the dependent variables capturing intended college majors, and who also indicated on the high school survey that there was at least a small chance that they would attend college in the future (we excluded 52 students who indicated that that is was extremely unlikely that they would attend college). Our analytic sample roughly mirrors characteristics of the district, as it is approximately 78% Hispanic, 12% Black, 7% white, and 3% other race/ethnicity (which includes Asian and Native American students). Additionally, approximately 87% of students in our sample qualified for either free or reduced lunch.

### Dependent variable

On the survey administered at the beginning of high school, students were asked to report how likely it is that they would choose to major in each of the following four STEM fields: biological sciences, physical sciences, computer science/technology and engineering. Response categories were on the following 5-point scale: 1 (not at all likely), 2 (somewhat unlikely), 3 (neutral), 4 (somewhat likely), to 5 (very likely). Exploratory analyses indicated that students' responses were correlated across certain STEM domains. Specifically, students who expressed a stronger likelihood or intention of majoring in computer science were also likely to express a strong intention of majoring in engineering (*r* = 0.6). A similar correlation was found between intentions to major in biological sciences and the physical sciences. Thus, we choose to ultimately collapse these four variables to create two dependent variables. Additionally, because responses were not normally distributed, we dichotomized them so that a score of 1 indicated that the student reported that they were either likely or very likely (a score of 4 or 5) to major in that field, vs. not likely (a score of 3 or below).

Thus, the first dependent variable for the analyses in this paper captures intentions to major in computer science or engineering (CS/E), distinguishing between those students who reported that were likely to major in either or both subjects (coded 1) vs. those who were not likely to major in either (coded 0). The second dependent variable captures intentions to major in either biological or physical sciences (B/PS), similarly distinguishing between those who were likely to major in either or both subjects (coded 1) vs not likely to major in either (coded 0). Across the entire sample, intentions to major in CS/E fields were quite high, with 46% of all students reporting that they were likely to major in such fields, and 54% reporting that they were not. In contrast, intending to major in B/PS fields was much less popular, with approximately 27% of all students reporting that they were likely to major in these fields, compared to 73% that were not^2^.

**Figure 1:**
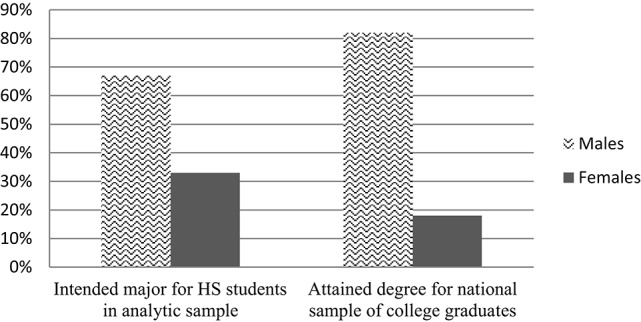
**Gender distribution of intended and attained degrees in computer science/engineering**. Source: National Science Foundation (2014). *Science and Engineering Indicators 2014*, (NSB 14-01). Arlington, VA

**Figure 2:**
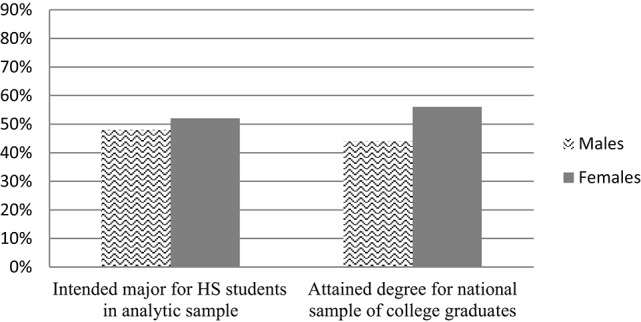
**Gender distribution of intended and attained degrees in biology/physical sciences**. Source: National Science Foundation (2014). *Science and Engineering Indicators 2014*, (NSB 14-01). Arlington, VA

Importantly, our data revealed substantial gender differences in these intentions. Of those students who were likely to pursue CS/E majors, approximately 67% of them were male, while only 33% were female. In contrast, among those expecting to major in B/PS majors, 48% were male and 52% were female. As seen in Figures 1, 2, we note that this gender breakdown is quite similar to the gender breakdown from national statistics on degree attainment in STEM fields (National Science Foundation, 2014), providing further support to the notion that gendered STEM expectations are strong precursors to subsequent patterns of inequality.

### Independent variables

#### Exclusionary and inclusionary peer beliefs and attitudes in the classroom

Our two key independent variables capture the beliefs and attitudes of peers in students' science classrooms, measured in the fall of students' 8th grade year and thus preceding our dependent variable by approximately a year. The first variable originated from the Michigan Study of Adolescent and Adult Life Transitions (MSALT) and is meant to capture biased or exclusionary beliefs (Eccles et al., 1990; Eccles and Harold, 1991; Ambady et al., 2001; Bleeker and Jacobs, 2004; Eccles, 2015). Students were asked the following question: “Who is better at science and math, girls or boys?” Students choose from 4 responses including “girls are better than boys,” “girls and boys are equally good,” “boys are better than girls,” or “I don't know.” Responses were dichotomized such that 1 represented the response “boys are better than girls” and 0 represented all other responses. Exploratory analyses confirmed that male peers were the likely source of biased beliefs, as approximately 2% of girls reported that boys were better than girls at math and science. We note that this is consistent with other studies of explicit gender stereotypes, which tend to find that girls are very unlikely to endorse such stereotypes (Schmader et al., 2004). Thus our final measure captures the proportion of boys in the science classroom who endorsed the belief that boys are better at math and science. Individual student responses from boys in the same science classroom were aggregated to create this classroom measure. The mean for this variable is approximately 0.16 indicating that on average, students in our sample spent their 8th grade year in science classrooms where 16% of boys held this gender/STEM bias.

Our second key independent variable is meant to capture inclusionary peer attitudes for girls in STEM fields, and has previously been used in the Trends in International Mathematics and Science Study (TIMSS) (Wilkins, 2004; Riegle-Crumb et al., 2011; Kastberg et al., 2013). Specifically, students were asked to report their level of confidence in their science ability by reporting how much they agreed with the statement “I usually do well in science.” Possible responses included “agree a lot,” “agree a little,” “disagree a little” and “disagree a lot.” Responses were dichotomized so that 1 = agree a lot and 0 = all other responses. Consistent with the notion that female peers can act as role models and support against gender stereotypes, we created a classroom measure of the proportion of females in the science classroom who were very confident (agreed a lot) in their ability to do well in science by aggregating the responses of individual female students. The mean for this variable is approximately 0.29 indicating that on average, students in our sample spent their 8th grade year in science classrooms where almost 30 percent of girls were very confident in their science ability.

#### Additional classroom variables

We include additional variables as classroom control variables in our analyses. The first of these is the percent of male students in the classroom who are very confident in their science ability, constructed in a parallel measure to that described above for our measure of female peer confidence. By including this measure we are able to better assess the unique contribution of having highly confident female peers vs. a general classroom climate where students are confident in their abilities. Our second classroom control variable captures differences across classrooms in students' science performance, measured as the average science grade earned by students (available from their academic transcripts) in the same classroom in their 8th grade year^3^.

To assess the association between different characteristics of classrooms, we calculated bivariate correlations. The association between male peer bias and female peer confidence was −0.11, indicating that although a higher percentage of biased male peers in the classroom was associated with a lower percentage of female peers with high science confidence, the magnitude of this relationship was very small in scope. The correlation between average classroom science grades and proportion of biased male peers was 0.05, while class grades and science-confident female peers were correlated at 0.20. Finally, the proportion of female peers with high science confidence also had a small positive correlation of 0.16 with the proportion of males with high science confidence. Thus in general the classroom characteristics we consider here appear to be quite independent of other.

#### Individual control variables

We also control on a number of characteristics to capture individual differences that may be relevant to intentions to major in STEM fields. First, we include measures for students' race/ethnicity, distinguishing between students who identified on the middle school survey as non-Hispanic white, Hispanic, Black, or other (which includes Asian and Native American students). A proxy for social class background was also included by utilizing a survey question asking students to estimate the number of books in their home. Responses on this variable range from 1 = few (0–10), to 4 = enough to fill several bookcases (more than 100). This survey item has been used in national studies of adolescents, as it is more reliable than asking students to report parent education level (Kastberg et al., 2013). An additional dichotomous variable captures whether or not the student has ever qualified for free or reduced lunch, as indicated on school administrative records. We also include a dichotomous measure from administrative records of whether the student was ever classified as Limited English Proficient (LEP).

Additionally, data from students' transcripts provided measures of students' academic background. This includes the final grade that the student earned in 8th grade science, as well as an indicator of the type of science class in which they were enrolled in 8th grade, where 1 = “Regular Science 8,” 2 = “Honors Science 8,” and 3 = “Other Science.” The third category includes a collection of several different courses that had different titles than the first two categories but were designated as science courses on the students' transcripts. Students' college expectations are captured by a dichotomous indicator taken from the high school survey, coded 1 for those who reported that they were very sure they will go to college and 0 for those who were not. Finally, we include measures from the 8th grade survey of students' own science confidence, utilizing the same measure described above to create the classroom measures. In analyses for male students (described below) we also include a measure of their own belief about whether or not boys are better at math and science.

Table 1 displays means (or proportions) and standard deviations for each of the independent variables in our analyses by gender. We note that female students have significantly higher science grades than male students (providing further evidence that a belief that boys are better at math and science is reflective of a stereotype and not a fact), as well as higher college expectations.

**Table 1:** **Descriptive statistics by gender**.

	**Females**		**Males**	
	**Mean**	***SD***	**Mean**	***SD***
**INDIVIDUAL CHARACTERISTICS**				
**Race/ethnicity**				
Black	0.13		0.11	
Hispanic	0.78		0.78	
Other race	0.02		0.04	
White	0.06		0.08	
**Family background**				
Books in the home	2.30	(1.09)	2.23	(1.06)
Free/reduced lunch	0.87		0.86	
Limited English proficient	0.29		0.30	
**Academic background**				
8th grade science confidence	0.30		0.34	
8th grade science grade	83.88	(6.45)	82.79	(7.34)
8th grade science course				
Science 8-regular	0.44		0.36	
Science 8-honors	0.43		0.42	
Other science	0.13		0.21	
College expectation	0.56		0.51	
**CHARACTERISTICS OF 8TH GRADE SCIENCE CLASS**				
Proportion of biased male peers	0.17	(0.24)	0.15	(0.19)
Proportion of confident female peers	0.29	(0.26)	0.29	(0.29)
Proportion of confident male peers	0.32	(0.31)	0.33	(0.26)
Average science grade	82.81	(4.47)	83.08	(4.84)
	*n* = 647		*n* = 626	

## Analyses and results

To examine whether exposure to biased male peers and confident female peers impacts the future intentions to major in either CS/E or B/PS majors (both of which are measured dichotomously), we utilize logistic regression analyses. We note here that our primary interest is in examining how peers shape the intentions of female students, and based on the findings of previous literature discussed earlier, we do not anticipate that our measures of peer attitudes and beliefs would predict male students' STEM intentions. However, we do run parallel models for male students in our sample and briefly discuss their results as a basis of comparison. Our models also include fixed effects for both the middle schools and high schools that students attend to ensure that the standard errors in the models are properly estimated (as students are clustered within schools) and that differences between schools are taken into account. All results reported are from two-tailed tests of significance.

Beginning with Table 2, Model 1 displays the results predicting whether or not female students intend to declare a computer science or engineering major. We present logistic regression coefficients (rather than odds ratios), to ease interpretation. Similar to the logic of linear regression models, negative coefficients indicate that as the independent variable increases, the outcome is less likely to happen, while positive coefficients indicate that an increase in the independent variable is associated with an increase in the likelihood of the dependent variable occurring. To illustrate the magnitude of variables of interest we then report predicted probabilities.

**Table 2:** **Logistic regression analyses predicting ***female students'*** likelihood of intending to major in different STEM fields**.

	**Model 1**		**Model 2**	
	**Computer science/engineering**		**Biological/physical sciences**	
	***B***	**SE**	***B***	**SE**
**CLASSROOM VARIABLES**				
Proportion of biased male peers	−0.877^*^	(0.440)	0.038	(0.437)
Proportion of confident female peers	0.703~	(0.439)	−0.243	(0.459)
Proportion of confident male peers	−0.088	(0.325)	−0.241	(0.342)
Average classroom science grade	−0.011	(0.031)	−0.030	(0.032)
**RACE/ETHNICITY (*****Ref*** = ***White*****)**				
Hispanic	−0.412	(0.483)	0.316	(0.471)
Black	−0.621	(0.524)	0.174	(0.503)
Other race	0.334	(0.680)	−0.560	(0.737)
**FAMILY BACKGROUND**				
Books in the home	0.203^*^	(0.095)	0.314^**^	(0.101)
Free/reduced lunch	1.229^**^	(0.441)	0.378	(0.389)
Limited English proficient	0.058	(0.224)	−0.108	(0.241)
**ACADEMIC BACKGROUND**				
8th grade science confidence	−0.035	(0.250)	0.736^**^	(0.250)
8th Grade science grade	0.000	(0.019)	0.036~	(0.020)
**8TH GRADE SCIENCE COURSE (*****Ref*** = ***Regular Science 8*****)**				
Honors science 8	0.030	(0.225)	0.210	(0.241)
Other science	−0.138	(0.410)	0.680~	(0.402)
College expectations	−0.005	(0.193)	0.248	(0.204)

*n = 647; Standard errors in parentheses*.

Two-tailed test: ^***^p < 0.001,

** *p < 0.01*,

* *p < 0.05, ~ p < 0.1*.

As seen in Model 1, there is a negative and statistically significant effect of male peer STEM bias on the likelihood of intending to major in a CS/E field. Specifically, as the percentage of biased males in girls' increases, the likelihood that they intend to declare a CS/E major in their future significantly decreases. Furthermore, female peer STEM confidence is positively associated with declaring a CS/E major, although we note that variable is only significant at the *p* < 0.10 level.

Regarding control variables in the model, the only significant associations with intentions of declaring of a CS/E major are found for measures of individuals' social class background. Specifically, reporting more books in the home is positively associated, as is qualifying for free and reduced lunch. This perhaps indicates a somewhat curvilinear relationship such that students from both high and low levels of social class background are inclined to pursue this field. Neither students' race/ethnicity nor measures of their academic background significantly predict CS/E intentions.

Model 2 displays parallel results for a model where the dependent variable is females' intentions to major in the biological or physical sciences. None of our focal peer measures, neither male peer bias nor female peer confidence, significantly predicts the likelihood of expecting to pursue these fields. Yet interestingly we note that while having more female peers who are very confident in their science ability does not predict B/PS intentions, a girl's own individual level of science confidence does predict such intentions. Results for control variables reveal that as with CS/E majors, there is a significant positive effect of having more books in the home. Also as before, race/ethnicity does not predict B/PS intentions. Finally we note that girls' science grades have a positive effect (*p* < 0.10) on their intentions to major in B/PS fields.

**Figure 3:**
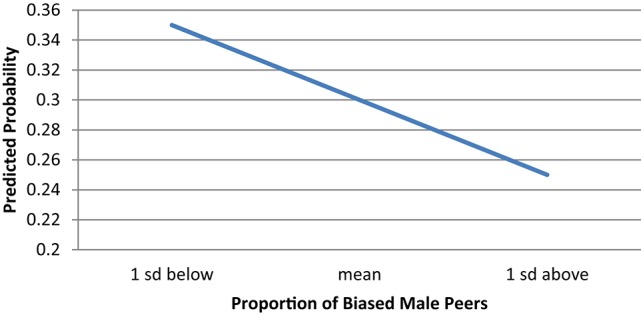
**Predicted probability of female students intending to declare a CS/E major by proportion of biased male peers**.

Thus we see evidence that peer characteristics that could be considered exclusionary (male bias) and inclusionary (female confidence) predict girls' future intentions to major in the very male-dominated fields of computer science and engineering, while they do not significantly predict girls' intentions to major in the equitable fields of the biological and physical sciences. To get a better sense of the magnitude of potential peer influence on girls' CS/E intentions, we calculated predicted probabilities holding everything else in the model at the mean and varying the level of our peer variables of interest. Beginning with male peer bias (shown in Figure 3), we see that when girls are in a classroom where the average level of bias is low (one standard deviation below the mean), their probability of intending to declare a CS/E major is approximately 0.35. But as the percent of boys in their science classroom who endorse gender bias increases to one standard deviation above the mean (approximately 40% of boys endorse stereotypes), girls' probability of declaring a CS/E major falls to only 0.25.

**Figure 4:**
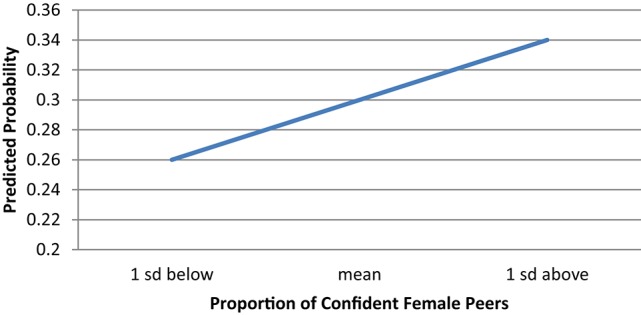
**Predicted probability of female students intending to declare a CS/E major by proportion of confident female peers**.

We similarly calculated predicted probabilities to assess the impact of exposure to highly confident female peers (shown in Figure 4). When girls are in a classroom with levels of female peer confidence that are one standard deviation below the mean, their predicted probability of intending to declare a CS/E major is about 0.26. As the percent of female peers with high levels of confidence increases to one standard deviation above the mean (about 60%), their probability of intending to pursue such a major increases to 0.34.

We now turn briefly to the results of parallel models run for boys in our sample. As seen in Table 3, none of our measures of peer characteristics significantly predict either boys' intentions to pursue CS/E majors (Model 1) or B/PS majors (Model 2). While it would perhaps be logical to assume that the presence of more male peers endorsing gender stereotypes that favor males (or even the presence of more highly confident male peers) might embolden or encourage boys' choices to pursue STEM fields, this is not the case. Indeed, we find no evidence that any characteristics of peers are associated with boys' choices to pursue either type of STEM field. For Model 1, we do find that relative to white students, Black male youth are significantly less likely to intend to pursue CS/E fields, while those students who qualify for free or reduced lunch, as well as those who expect to go to college, are significantly more likely to intend to enter CS/E fields. Having high levels of confidence in their own science ability does significantly predict male students' intentions to declare a BP/S major, while net of other factors in the model, higher science grades negatively and significantly predict such intentions. No other control variables were significant predictors of future STEM plans.

**Table 3:** **Logistic regression analyses predicting ***male students'*** likelihood of intending to major in different STEM fields**.

	**Model 1**		**Model 2**	
	**Computer science/engineering**		**Biological/physical sciences**	
	***B***	**SE**	***B***	**SE**
**CLASSROOM VARIABLES**				
Proportion of biased male peers	−0.067	(0.547)	−0.805	(0.661)
Proportion of confident female peers	−0.081	(0.331)	−0.032	(0.368)
Proportion of confident male peers	−0.345	(0.418)	−0.106	(0.461)
Average classroom science grade	−0.023	(0.028)	0.027	(0.032)
**RACE/ETHNICITY (*****Ref** = **White*****)**				
Hispanic	−0.662	(0.406)	−0.106	(0.411)
Black	−1.165^*^	(0.471)	−0.057	(0.49)
Other race	0.219	(0.588)	0.402	(0.546)
**FAMILY BACKGROUND**				
Books in the home	−0.003	(0.089)	0.032	(0.100)
Free/reduced lunch	0.701^*^	(0.327)	−0.035	(0.341)
Limited English proficient	0.255	(0.211)	−0.102	(0.247)
**ACADEMIC BACKGROUND**				
8th grade science confidence	0.211	(0.225)	0.808^***^	(0.244)
8th grade gender bias	0.087	(0.277)	−0.041	(0.330)
8th grade science grade	−0.013	(0.016)	−0.043^*^	(0.018)
**8TH GRADE SCIENCE COURSE (*****Ref*** = ***Regular Science 8*****)**				
Honors science 8	0.360	(0.226)	−0.272	(0.258)
Other science	0.712^*^	(0.329)	0.091	(0.352)
College expectations	0.589^**^	(0.187)	0.241	(0.211)

*n = 626; Standard errors in parentheses*.

Two–tailed test:

*** *p < 0.001*,

** *p < 0.01*,

* *p < 0.05, ~ p < 0.1*.

## Discussion

Building on the insights of prior psychological and sociological research on the power of local environments to shape gendered outcomes in STEM fields, the goal of this study was to explore how peers could be the source of both exclusionary and inclusionary messages in the classroom. Specifically, we considered the potential influence of both biased male peers and confident female peers on shaping the future STEM intentions of adolescent females. In doing so, our study makes a new contribution to the field by directly measuring both positive and negative peer perspectives in the actual science classrooms that young people inhabit, as well as focusing on the critical stage of adolescence when future plans begin to firmly materialize (Morgan et al., 2013). Additionally our study advances prior research by distinguishing between intentions to pursue the male-dominated fields of computer science and engineering, compared to the more gender equitable fields of the biological and physical sciences (National Science Foundation, 2014).

Results of multivariate longitudinal analyses of our sample of diverse youth suggest that peers may indeed be important sources of both exclusionary and inclusionary messages that are relevant in shaping girls' STEM intentions. Specifically, we found that exposure to a higher percentage of 8th grade male peers in the classroom who endorsed explicit gender/STEM stereotypes significantly and negatively predicted girls' later intentions to pursue a computer science/engineering major. Yet conversely, exposure to a higher percentage of confident female peers in the science classroom positively predicted such intentions. These results were specific to CS/E majors, as we did not find similar effects for intentions to major in B/PS fields. This suggests that in contrast to fields that are quite normative for females to enter, peers are an important source of messages regarding whether or not girls should pursue non-traditional STEM fields.

Additionally, we note that the effects of male peer bias and female peer confidence are largely independent of one another, as we noted earlier the presence of a very small correlation (−0.11), and exploratory analyses also revealed there was not a significant interaction between the two. To the extent that these peer effects are additive and of similar magnitude, this suggests that the negative effect of a classroom where a high percentage of boys endorsed gender stereotypes could be counteracted by a similarly high percentage of very confident female peers. Yet if such highly confident female peers were absent, the negative effect of boys' bias could indeed substantially dampen girls' intentions to pursue CS/E fields. On the other hand, in the absence of bias from male peers, a classroom context characterized by a high percentage of confident female peers could lead to a noteworthy increase in girls' plans to pursue these male-dominated fields. Thus, our study calls attention to the importance of examining both positive and negative sources of influence within local contexts, as well as highlights the need for more research that focuses on male peers in particular. We argue that efforts to increase female representation in CS/E fields need to pay more attention to understanding the attitudes, beliefs, and choices of boys, including how they may directly or indirectly shape girls' attitudes, beliefs, and choices.

Further, our study is also somewhat unique regarding the predominantly Hispanic composition of our adolescent sample, a population that is increasing dramatically in the U.S. yet still often under-represented in research. Yet we also note that gendered patterns of interest in pursuing CS/E and B/PS fields among our mostly minority sample closely mirror national levels of gendered attainment in college degrees in these fields. This is consistent with research that finds very similar patterns of male advantage across racial/ethnic groups (Riegle-Crumb and King, 2010). Additionally, in exploratory analyses we tested for but did not find evidence of significant interactions between students' race/ethnicity and our peer variables of interest. We concur with feminist scholars who call for the need for more research that considers where and how the intersection of gender with race/ethnicity shapes individual trajectories, including those in STEM fields; this entails more research that seeks to compare the experiences of women from different backgrounds, as well as research that explicitly focuses on giving voice to the obstacles and experiences of minority women (Browne and Misra, 2003; Ong, 2005; Carlone and Johnson, 2007).

## Limitations

Finally, we note that while our study addresses some of the limitations of prior research, it is nevertheless subject to its own limitations. Most notably our data contains no information regarding whether or not girls are aware of the views of their peers, either the negative stereotypes endorsed by their male peers or the positive and confident views of their female peers. Therefore while our study moves past the confines of some experimental research by measuring phenomenon that are indeed present in actual classrooms, we cannot test how this influences tangible experiences and interactions. Ideally, future research should combine explicit measurement of what peers think (as we have done) with measures of what individuals believe that their peers think, as well as measures of actual classroom experiences and interactions. Such a research design would provide researchers with a remarkably rich picture of the local context of STEM classrooms that can have strong implications for how young girls view their possible and desired futures.

## Implications

Our study calls attention to the importance of examining both positive and negative sources of influence within local contexts, as well as highlights the need for more research that focuses on male peers during the formative stages of adolescence in particular. Recent studies have found that adult men in STEM fields in both the academy and industry are prone to endorse gender stereotypes regarding women's innate abilities, and that such views can be linked to subsequent discrimination (Bobbitt-Zeher, 2011; Ecklund et al., 2012). Yet the extant empirical research on gender inequality at earlier stages in STEM trajectories focuses almost exclusively on changing girls' attitudes and choices, with correspondingly little attention to examining the boys with whom they share classrooms and schools on a daily basis. We therefore argue that more research should examine boys' beliefs about gender and how such views are linked to exclusionary behavior and interactions, with an ultimate eye towards creating new programs and interventions that attend to boys' role in creating and sustaining inequality in certain STEM fields.

## Ethics statement

This study has been processed by the Office of Research Support at the University of Texas at Austin and was determined as “Exempt” from IRB review because it entails secondary data analyses.

## Author contributions

CR developed the theoretical framework, designed the study, conducted most of the data analyses, and wrote the majority of the text. KM assisted with the theoretical framing, constructed variables, and assisted with data analyses.

### Conflict of interest statement

The authors declare that the research was conducted in the absence of any commercial or financial relationships that could be construed as a potential conflict of interest.

## Footnotes

^1^The category of physical science generally includes both chemistry and physics. Although the latter remains predominantly male, chemistry is indeed gender equitable (with 50% of undergraduate degrees in the field awarded to women) and is also the much larger category (National Science Foundation, 2014). Specifically, of those earning degrees in the category of physical science, over 2/3 of them were in chemistry (National Science Foundation, 2014).

^2^Consistent with our decision to treat these as separate fields, we note that there was little overlap between students intending to major in CS/E and B/PS fields. Specifically only 12% of female students and 18% of male students were coded as 1 on both variables.

^3^In exploratory analyses, we also included classroom averages of students' social class background as measured by students' reports of books in the home (included as an individual level control), as well as the gender composition of the classroom (which had very little variation as most classes were comprised of equal numbers of male and females). These variables were not statistically significant and their inclusion did not alter any of our key findings. Therefore for the sake of parsimony they are not included in the final models.
